# Public health information for minority linguistic communities

**DOI:** 10.2471/BLT.21.285617

**Published:** 2021-11-17

**Authors:** Pierpaolo Di Carlo, Bradley McDonnell, Lisa Vahapoglu, Jeff Good, Mandana Seyfeddinipur, Katarzyna Kordas

**Affiliations:** a Department of Linguistics, University at Buffalo, 609 Baldy Hall, 14260 Buffalo NY, United States of America (USA).; b Department of Linguistics, University of Hawai’i at Mānoa, Mānoa, USA.; c RENEW Institute, University at Buffalo, Buffalo, USA.; d Endangered Languages Documentation Programme, Berlin-Brandenburg Academy of Sciences and Humanities, Berlin, Germany.; e Department of Epidemiology and Environmental Health, University at Buffalo, Buffalo, USA.

*Crisis and emergency risk communication* guidelines^1^ stress that the success of a communication campaign is determined by how well its design reflects the diversity of the intended audience. Of all the options of customizing communications, translating messages into the languages that are relevant to diverse audiences is the most crucial, as this determines their reach. However, expense and time considerations tend to limit the linguistic diversity of communication campaigns to majority languages.

The crisis and emergency risk communication approach relies on the assumption that speakers of minority or marginalized languages will understand messages delivered in a major language. We do not question this assumption. Rather, we want to raise awareness that approaches based on this assumption overlook how language choice influences the message’s reception. In many minority or marginalized groups around the world, using the language of the majority may evoke histories of domination and exclusion, which would have a negative influence on the perceived trustworthiness of the communicator. Here we argue that in exceptional circumstances such as the coronavirus disease 2019 (COVID-19) pandemic and associated infodemic (when too much information, including false and misleading, is widely available), communicating to linguistically diverse audiences is best achieved by using different groups’ native languages and communicative styles. Reaching this ambitious goal is more realistic today than it was two decades ago. 

## Many translations equal success?

**Fig. 1:**
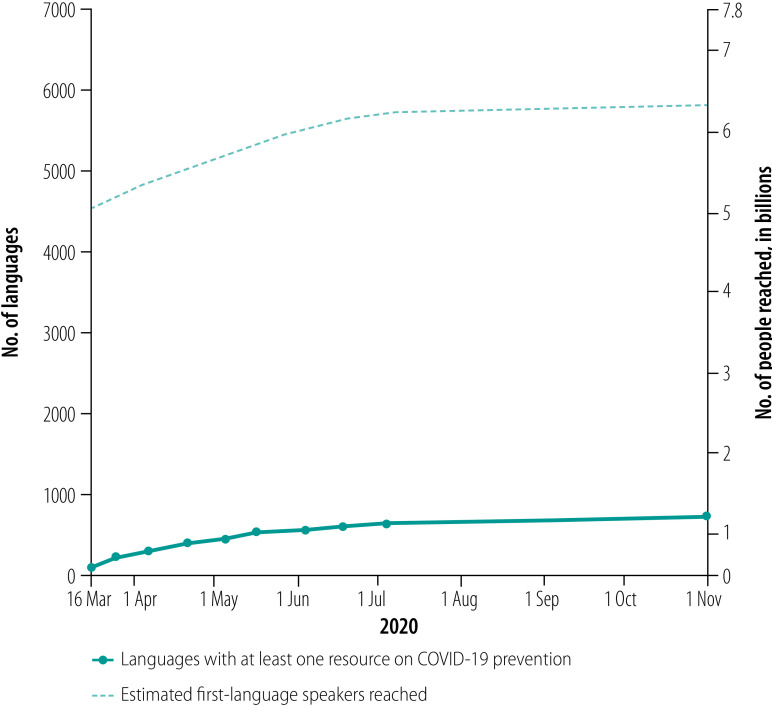
Number of languages in which COVID-19 preventive measures have been translated and number of speakers reached, 2020

Throughout 2020, efforts by governments, nongovernmental organizations, academics and communities have resulted in the translation of COVID-19 preventive measures in approximately 700 languages.^2^ About 630 of these are minority or marginalized languages (Fig. 1).

COVID-19: coronavirus disease 2019.

Note: Numbers start from the first count of the resources made on 16 March 2020 (= 110 languages).^2^ At that time, translations of COVID-19 preventive measures were already available in approximately 50 major languages. On 1 November 2020, the languages with at least one resource on COVID-19 prevention were about 700. Estimated speakers reached are based on available figures for first-language speakers.^3^ Both data sets are estimated.

Data source: Eberhard DM et al., 2020.^3^

Based on the estimated 7000 languages currently spoken in the world and their documented speaker populations,^3^ native speakers of the remaining 6300 languages or so, which are minority or marginalized, are about one tenth of the world’s population. Since most of these populations are likely proficient in one of the major languages of the world, policy-makers would assume that existing translations of COVID-19 preventive measures are understandable by the near totality of the global population. By this metric, the translation efforts made thus far appear to be a substantial success. Upon closer analysis, however, we believe that this would be a mistaken assumption.

## Infodemic affects minorities

The evolution of the COVID-19 pandemic was accompanied by an unprecedented circulation of unfounded information, which made it difficult for people to know what or whom to trust. Such an infodemic is particularly concerning for minorities and marginalized communities worldwide who have experienced oppression and stigmatization and who, as a result, are historically more prone to distrust state institutions and governments.^4^ In the crisis and emergency risk communication context, broad distrust in authorities and health communicators makes risk communication less effective. Preventive measures such as social distancing, wearing personal protective equipment and restricting travel may not achieve their intended public health goal because the public may decide not to adopt them. Failure to communicate effectively with the public is particularly consequential in countries with weak health systems, that is, countries in which the response to COVID-19 is primarily one of behaviour change prevention efforts because limited financial resources restrict vaccination and treatment options. Most of the world’s marginalized languages are spoken in these countries. Thus, ineffective public health communication about COVID-19 is likely to disproportionately affect populations that speak marginalized languages.

## Rebuilding public trust

We argue that the challenge posed by communicating risk during the pandemic – and similar situations – is not simply one of translation but of (re)building public trust in those who provide information, especially in countries or communities with weak health systems. From a linguistic perspective, we wish to emphasize two main considerations. First, research in psycholinguistics, neurolinguistics and marketing indicates that messages received in a person’s first or native language are likely to trigger a stronger emotional response than messages in this person’s second language,^5^^,^^6^ thus making the content both more memorable^7^ and more likely to result in actions and changes in behaviour.^8^ Second, for many of the 630 minority and marginalized languages mentioned above, existing COVID-19 information resources consist of only one or two written or multimedia documents that are verbatim translations from the World Health Organization (WHO) and other official sources. Few of these existing translations in marginalized languages appear to be designed to cultivate trust among the message recipients.

The first consideration suggests that, in the context of the current infodemic, producing and disseminating messages in a language that people understand but do not identify with may reduce effectiveness or even have an adverse effect on how the message is received and acted upon, especially by minorities and marginalized groups. To counteract the lack of trust and potential ineffectiveness of COVID-19 prevention messages, we call for additional translation efforts aiming to disseminate information in all the 7000 languages of the world. Even minor outbreaks can contribute to the ongoing threat posed by the development of new variants of the virus. The second consideration above suggests that simply being exposed to a message in one’s first language may be insufficient for its acceptance. Following *Crisis and emergency risk communication *guidelines,^1^ health messages should both use the local language according to local styles of delivery, and be designed to leverage audience-specific features promoting trust. This approach can be implemented through, for instance, associating messages with well known and esteemed members of the target community. 

## Leveraging existing networks 

Producing public health messages in thousands of languages and associated with trusted community members would have been impossible until recently. However, over the past three decades concern for the documentation of endangered languages has been growing,^9^ and this new development has brought increasing numbers of linguists into direct contact with marginalized communities. Many of these linguists are also committed to finding ways to create reciprocal benefit for their host communities.^10^ These collaborative networks open significant opportunities for partnerships between transdisciplinary teams, including linguists and specialists in public health as well as marginalized and vulnerable communities, to co-create and transmit key public health messages. This model enables the generation of messages that account for preferences in language, cultural norms, nuance in communication and current trends in knowledge or misinformation. A case in point is the virALLanguages initiative.^11^^,^^12^

## VirALLanguages

Of the many commendable translation projects responding to the COVID-19 pandemic, virALLanguages is unique for three reasons. First, it was developed through the transdisciplinary collaboration of documentary linguists, public health specialists and social scientists, who co-created virALLanguages with members of communities speaking marginalized languages. Second, its design gives priority to (re)building public trust in those providing information through the production of multimedia messages by local teams featuring spokespeople who are well known and esteemed by the community. Third, native speakers are trained and encouraged to actively create original messages rather than produce verbatim translations of pre-existing standardized messages. Native speakers are required to read a five-page reference text and pass a comprehension test before creating messages. This reference text is designed and continuously updated by public health and other experts volunteering for virALLanguages, and it includes the most current information from WHO and the United States Centers for Disease and Control Prevention. The text is not limited to the illustration of COVID-19 preventive measures and contains general information that would allow native speakers to have an improved understanding of the main public health issues at stake. This training process was aimed at providing native speakers with enough information so that they could produce messages that were both culturally appropriate and accurate.

The networks activated by linguists enabled even remote communities to be reached. Within a few months, local teams of volunteers created more than 90 audio and video resources (1 to 5 minutes long) in 47 minority or marginalized languages of Cameroon, Indonesia and Pakistan. A systematic protocol was followed to ensure content accuracy, and resources were made available through social media outlets and streaming platforms as playable files. In some cases, messages were also broadcast by local radio stations. Due to lack of funding for this volunteer-run project, it was impossible to systematically evaluate the public health impact of the resources created in the initial phase of the project or to scale it up to additional languages. However, anecdotal reports were positive and in some cases, explicitly connected community interest in the messages to the fact that they were presented in a local language by individuals that community members personally trusted.

Weak health systems and pre-existing health disparities exacerbate the COVID-19 pandemic, which will remain a worldwide emergency for the foreseeable future. Public health communication can help overcome this crisis only if substantial effort is made to increase its effectiveness through leveraging the world’s linguistic diversity and improving on existing models such as the virALLanguages initiative. Doing so is now an ambitious but attainable goal, thanks to recent advances in linguistic field-based research and communications technology.
